# Classifying cGAS-STING Activity Links Chromosomal Instability with Immunotherapy Response in Metastatic Bladder Cancer

**DOI:** 10.1158/2767-9764.CRC-22-0047

**Published:** 2022-08-04

**Authors:** Mateo Sokač, Johanne Ahrenfeldt, Kevin Litchfield, Thomas B.K. Watkins, Michael Knudsen, Lars Dyrskjøt, Martin R. Jakobsen, Nicolai J. Birkbak

**Affiliations:** 1 Department of Molecular Medicine, Aarhus University Hospital, Aarhus, Denmark.; 2 Department of Clinical Medicine, Aarhus University, Aarhus, Denmark.; 3 Bioinformatics Research Center, Aarhus University, Aarhus, Denmark.; 4 Tumour Immunogenomics and Immunosurveillance Laboratory, University College London Cancer Institute, London, United Kingdom.; 5 Cancer Evolution and Genome Instability Laboratory, The Francis Crick Institute, London, United Kingdom.; 6 Department of Biomedicine, Aarhus University, Aarhus, Denmark.

## Abstract

The cGAS-STING pathway serves a critical role in anticancer therapy. Particularly, response to immunotherapy is likely driven by both active cGAS-STING signaling that attracts immune cells, and by the presence of cancer neoantigens that presents as targets for cytotoxic T cells. Chromosomal instability (CIN) is a hallmark of cancer, but also leads to an accumulation of cytosolic DNA that in turn results in increased cGAS-STING signaling. To avoid triggering the cGAS-STING pathway, it is commonly disrupted by cancer cells, either through mutations in the pathway or through transcriptional silencing. Given its effect on the immune system, determining the cGAS-STING activation status prior to treatment initiation is likely of clinical relevance. Here, we used combined expression data from 2,307 tumors from five cancer types from The Cancer Genome Atlas to define a novel cGAS-STING activity score based on eight genes with a known role in the pathway. Using unsupervised clustering, four distinct categories of cGAS-STING activation were identified. In multivariate models, the cGAS-STING active tumors show improved prognosis. Importantly, in an independent bladder cancer immunotherapy-treated cohort, patients with low cGAS-STING expression showed limited response to treatment, while patients with high expression showed improved response and prognosis, particularly among patients with high CIN and more neoantigens. In a multivariate model, a significant interaction was observed between CIN, neoantigens, and cGAS-STING activation. Together, this suggests a potential role of cGAS-STING activity as a predictive biomarker for the application of immunotherapy.

**Significance:**

The cGAS-STING pathway is induced by CIN, triggers inflammation and is often deficient in cancer. We provide a tool to evaluate cGAS-STING activity and demonstrate clinical significance in immunotherapy response.

## Introduction

Over the past decade, the application of immune checkpoint inhibitors (CPI) against immune checkpoints PD-1/PD-L1 and CTLA-4 have revolutionized cancer treatment, both increasing cancer survival time and leading to complete remission in a significant proportion of patients (1). Indeed, CPI therapy is now standard of care in metastatic melanoma and non–small cell lung cancer, and its proficiency demonstrated in multiple other cancer types including bladder cancer (BLCA; ref. 2). Immunotherapy is also being evaluated in ovarian cancer; however, this remains restricted to clinical trials with only modest response rates ranging from 4% to 15% (3, 4). The mechanism of action of CPI therapy is based upon blocking of inhibitory signals transmitted from cancer cells to infiltrating immune cells (5) through the PD-L1 and CTLA-4 checkpoints, which are commonly upregulated in cancer cells. This therapeutic blockade of inhibitory signals from the cancer releases the host immune system to unleash an immune response directed against cancer neoantigens. Neoantigens arise during tumor development through the introduction of random mutations into protein-coding genes leading to the generation of novel peptides with antigenic potential. These are the likely targets of an anticancer immune response, and clinical trials have demonstrated that patients where the cancer shows a high expression of PD-L1 and/or harbors a high number of mutations generally respond better to CPI therapy (6, 7). However, conflicting results are emerging and patients with low tumor mutation burden (TMB) also show response in some cases (8). Indeed, an increasing volume of recent work indicates that additional layers of immune evasion are present in cancer, suggesting that blocking neoantigen activation of T cells through the PD-1/PD-L1 axis may only represent one angle of a complex immune escape machinery, and approaches amending TMB with immune system activation have been proposed (9).

Ayers and colleagues demonstrated how an inflamed T-cell gene expression signature significantly predicted response to CPI therapy in multiple types of cancer, independently of both PD-L1 status and TMB (9, 10), suggesting that tumor suppression of infiltrating immune cells may represent a discrete mechanism of immune evasion. To elucidate the potential mechanisms of immune suppression, work from Rosenthal and McGranahan demonstrated how immunoediting, disruption to the antigen-presenting machinery, and epigenetic silencing of selected genes are associated with reduced immune infiltration into lung cancer tumors (11, 12).

Recent clinical trials in lung cancer have shown how the addition of a short regimen of chemotherapy or radiotherapy significantly improves the response rates to CPI (13–15). Both chemotherapy and radiotherapy induce DNA damage, chromosomal instability (CIN), and cell death, which stimulate an innate immune response through activation of the cGAS-STING pathway. When functioning properly, cGAS (cyclic GMP-AMP synthase) directly binds cytosolic DNA and catalyze the production of the small messenger molecule 2′3′ cGAMP, which in turn binds to and activates STING1 (stimulator of interferon genes). STING1 itself induces signaling pathways that release type 1 IFNs, through activation of TBK1 and IRF3, and inflammatory cytokines through both NFκB and JAK/STAT signaling, resulting in an influx of immune cells to the local tissue. In cancer, an expanding body of evidence implicates disruption of the STING1-mediated IFN response with immune escape, resulting instead in noncanonical activation of the NFκB pathway downstream of STING1, and in downregulation of homologous recombination through PARP1 (16–18).

CIN is a hallmark of cancer, with most solid tumors demonstrating some level of aneuploidy. CIN leads to cytosolic DNA and may itself induce an inflammatory response through STING1 activation (16). It is likely that tumors with high levels of ongoing CIN, where disruption to the chromosomal context is common, may select for reduced or abnormal STING1 activation as they evolve. Furthermore, disrupted STING signaling itself may further increase CIN particularly acting through the cell cycle (19, 20), further reducing inflammatory signals within the tumor microenvironment and thereby reducing the effect of both chemotherapy and CPI therapy. Thus, knowing the level of activation of the cGAS-STING pathway within a tumor prior to therapy administration may help inform therapy response. Unfortunately, no definitive model exists to determine the activation status of the cGAS-STING pathway, but pathway activation has previously been investigated using gene expression–based methods (21). Particularly, Della Corte and colleagues (20) recently demonstrated a three-gene clustering approach to identify STING1 activation based on cGAS, CCL5, and CXCL10 associated with DNA damage and immune activation in patients with treatment-naïve lung cancer. However, while simple to apply, this signature depends on hierarchical clustering of only three genes, and as such may be prone to random gene fluctuations and may be difficult to apply to single samples.

We hypothesize that CIN in tumors with functional cGAS-STING pathway creates an inflammatory environment rich in immune cells. When these tumors also present targetable neoantigens, which are associated with high TMB, they are particularly sensitive to checkpoint immunotherapy.

In this work, we specifically investigate the interaction between the cGAS-STING pathway, tumor inflammation, CIN, TMB, and response to immunotherapy. Using expression patterns of known cGAS-STING interacting genes, we define a gene expression signature of cGAS-STING pathway activation using a machine-learning approach. We then apply this signature to whole-exome sequence (WES) data and paired RNA sequencing (RNA-seq) data from patients with advanced BLCA receiving immunotherapy. Here, we demonstrate how the combination of CIN, TMB, and cGAS-STING activation status is able to stratify patients into subgroups with predictive and prognostic relevance.

## Materials and Methods

### The Cancer Genome Atlas Data Acquisition and Processing

To capture diverse mechanisms of cGAS-STING pathway activation, tumor data were obtained from a total of 2,307 samples representing five diverse cancer types from The Cancer Genome Atlas (TCGA): BLCA, lung adenocarcinoma (LUAD), lung squamous cell carcinoma (LUSC), skin cutaneous melanoma (SKCM), and ovarian cancer. The cancer types were chosen to create a balanced but manageable dataset that included commonly immunotherapy-treated cancer types; BLCA, LUAD, LUSC, SKCM, but representing different mechanisms of carcinogenesis and of immune escape. Specifically, BLCA is commonly treated with Bacillus Calmette Guerin immunotherapy, believed to trigger local inflammation through an innate immune response (22, 23), aiming to trigger local inflammation through innate immune response (23). LUAD and LUSC are both primarily smoking induced lung cancers and both demonstrate good immunotherapy responses, particularly among patients with a high mutation burden and PDL1 expression (22) Never smokers almost exclusively develop LUAD (24), indicating different carcinogenic mechanisms at play. SKCM is predominantly induced by UV light, and is characterized by a large mutation burden. These cancers are aggressive, but fortunately respond well to checkpoint immunotherapy (25). In addition, we included ovarian cancer, which is not commonly treated with immunotherapy, to reflect a cancer type which is driven primarily by CIN (26), a known trigger of inflammation (27). We collected the uniformly normalized RNA expression data for all samples from the UCSC Xena database (28). Clinical, SNP and mutation data were obtained from TCGA genomic data portal (https://portal.gdc.cancer.gov). Affymetrix SNP 6.0 profiles were obtained for paired tumor–normal samples and processed as described previously (29) using ASCAT (30) version 2.4.2 to obtain allele-specific copy number, purity, and ploidy estimates.

### The BLCA Immunotherapy Cohort Acquisition and Processing

Tumor molecular and response data were obtained for 348 patients with BLCA treated with immunotherapy from Mariathasan and colleagues (31). A subset of the patients was previously pretreated with platinum chemotherapy. RNA-seq data were aligned against hg38 using STAR (32) version 2.7.2 and processed to generate count and transcript per million (TPM) expression values with Kallisto (33) version 0.46.2. WES data were processed using GATK (34) version 4.1.5 and ASCAT version 2.4.2 to obtain mutation and allele-specific copy number, purity, and ploidy estimates.

### Unsupervised Clustering with Uniform Manifold Approximation and Projection

TPM expression data from 2,307 TCGA samples representing BLCA, LUAD, LUSC, SKCM, and ovarian cancer were scaled and centered around zero, Then the expression of eight genes (CCL5, CXCL11, CXCL10, CXCL9, CGAS, IFI16, ATM, STING1) was analyzed using Uniform Manifold Approximation and Projection (UMAP; ref. 35) using the following parameters: n_neighbours = 30, min_dist = 0.01, spread = 0.05, learning_rate = 0.01, n_components = 2. To identify robust clusters, the two-dimensional UMAP output was analyzed with k-means clustering for *K* = 1–8. The optimal value of *K* was defined as *K* = 4 based on the elbow method. Using this method, we clustered all samples into four cGAS-STING groups (CSG).

### Summary Measures

We defined the level of immune cell infiltration using the tumor-infiltrating leukocyte (TIL) score established by Danaher and colleagues (36) based on whole tumor RNA-seq data, implemented as described previously (12). CIN was defined on the basis of the weighted genome integrity index (wGII), defined as described previously (37). TCGA TMB was calculated as the number of nonsynonymous mutations per megabase. Tumor neoantigens and immune cell infiltration in the BLCA immunotherapy cohort was obtained from the original publication (31).

### Defining a cGAS-STING Prediction Model Using Random Forest Model

To build a prediction model that recaptures the UMAP clusters, we defined a training and test subset of TCGA RNA-seq data containing 80% and 20% of total data, respectively. On the training data, we applied random forest using 200 trees and validated the results on the testing subset. The model showed out-of-bag error of 7.85% on training data and similar results for testing data. To classify the test cohort into CSGs, this random forest model was applied to expression data normalized by centering the values of each gene, then dividing by the SD.

### Data and Software Availability

Code to run CSG calling along with TCGA IDs, cancer types and CSG clusters is available at https://github.com/mxs3203/csg_prediction

## Results

### Genes Involved in the cGAS-STING Pathway Cluster Samples into Distinct Immune Groups

**FIGURE 1:**
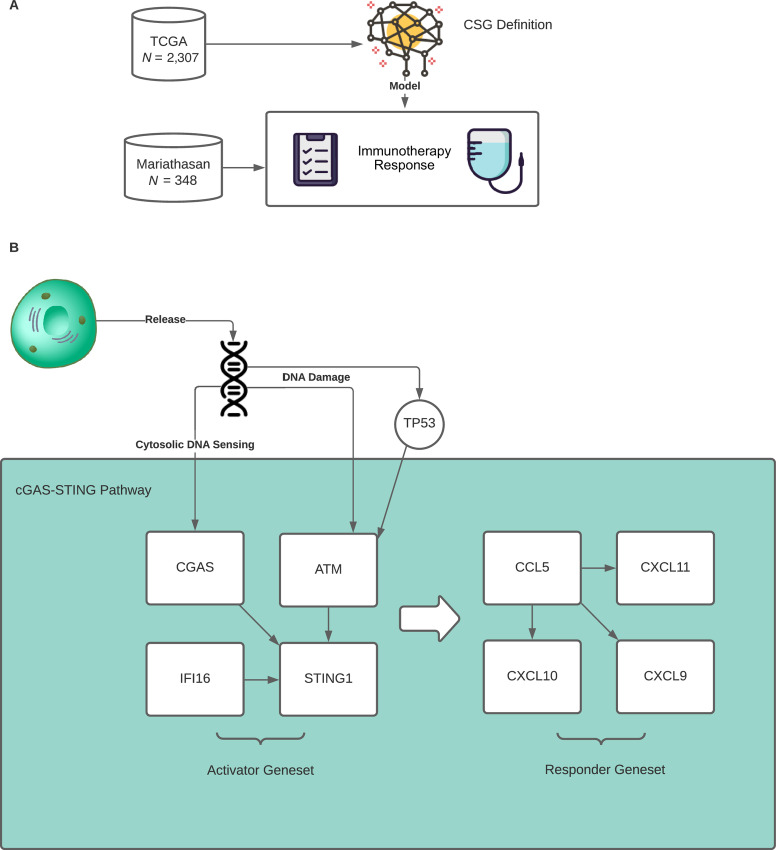
Study overview. **A,** The study utilized 2,307 TCGA tumor samples representing five distinct cancer types (bladder, lung adenocarcinoma, lung squamous, melanoma, and ovarian cancer) to define CSGs of differential immune activation based on expression of cGAS-STING genes. This signature was then tested in an independent dataset of patients with metastatic BLCA (Mariathasan cohort) treated with checkpoint immunotherapy for predictive and prognostic relevance. **B,** Grouping of cGAS-STING pathway genes split into two gene sets, Activators and Responders, based on their function in the pathway.

**FIGURE 2:**
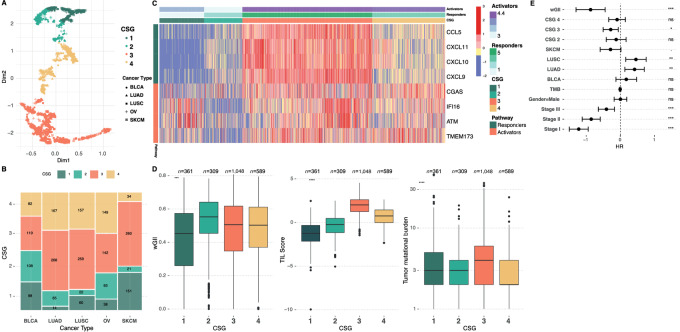
CSGs in TCGA. **A,** UMAP projection of TCGA samples into two-dimensional space. Samples are clustered using k-means into CSGs represented by distinct colors. **B,** Distribution of CSG groups within the cancer types. **C,** Heatmap showing the expression of the individual Activator and Responder genes, samples clustered by CSG. Annotation bars represent the mean values of genes summarized by Activator or Responder labels. **D,** Boxplots showing wGII (a CIN measure), TILs, and TMB, by CSG. **E,** Multivariate Cox proportional hazards model including CSG, wGII, TMB, stage, and cancer type as covariates, showing improved outcome in CSG-3.

To investigate the potential role of cGAS-STING pathway activation in response immunotherapy and its association with CIN, we endeavored to develop a model based on the expression levels of genes with known involvement in the cGAS-STING pathway (Fig. 1A). Through literature search and known biological evidence from studies exploring the cGAS-STING pathway activation in various immune cell types, we selected 20 genes with a previously reported role in the innate immune response and involvement with the cGAS-STING pathway (Supplementary Table S1). On the basis of gene expression data obtained from TCGA, we observed that a set of eight genes showed high intersample variation (SD > 1) and robust expression levels (average expression > cohort median), while representing distinct parts of the cGAS-STING pathway (Supplementary Fig. S1). From these, we defined an “activator” gene set involved in pathway initiation (STING1, cGAS, IFI16, and ATM), and a “response” gene set observed to increase following pathway initiation (CCL5, CXCL9, CXCL10, and CXCL11; Fig. 1B). Next, we asked whether the expression levels of these genes may cluster the samples into distinct groups, potentially informing on cGAS-STING pathway activation. To investigate this, we performed unsupervised clustering using UMAP (35) on 2,307 samples from TCGA, representing diverse cancer types commonly treated with immunotherapy (BLCA, LUSC, LUAD, SKCM), and cancers driven by CIN (ovarian cancer). The UMAP output was further divided into clusters by k-means, with the optimal number of clusters (*K* = 4; Supplementary Fig. S2). This shows that the expression level of the identified biomarker genes can stratify tumor samples into four distinct groups, here named CSGs 1–4, potentially indicating different levels of activation of the immune system (Fig. 2A). CSG sizes in TCGA and in the BLCA immunotherapy cohort can be found in Supplementary Fig. S3. These four identified groups were found in all five cancer types, but showed different distributions within each (Fig. 2B). Particularly, we observe that CSG-3 is dominant in LUAD, LUSC, and SKCM where it represents more than 50% cases. Conversely, we observe that in BLCA and ovarian cancer, CSG-3 represents a smaller fraction of cases, while CSG-1 and CSG-2 are increased. In addition, within ovarian cancer the highest fraction of CSG-4 is found.

### CSG Associate with Differential Activation of the cGAS-STING Pathway

To understand the biological differences driving the CSG, we compared the expression of the individual CSG genes between the groups (Fig. 2C). We observe that CSG-1 and CSG-2 are defined by low expression of both activator and response genes relative to CSG-3 and CSG-4. Comparing CSG-1 and CSG-2, CSG-1 shows relatively higher expression of the activator genes, and relatively lower expression of the response genes (Supplementary Fig. S4A and S4B). This indicates that group 1 has a functional but not activated pathway. Conversely, CSG-2 shows increased expression of response genes, but low expression of activator genes indicating a nonfunctional pathway. CSG-3 and CSG-4 are characterized by higher overall expression of the cGAS-STING genes, with CSG-3 showing the highest level of expression of all genes, indicating full activation of the pathway. Conversely, CSG-4 shows lower expression of the response genes suggesting activation of the cGAS-STING pathway but failure to activate the downstream IFN signaling cascade designed to produce IFNs and attract tumor-infiltrating Leukocytes (TIL). This is further supported by CSG-4 demonstrating lower levels of TILs relative to CSG-3 (Supplementary Fig. S4C). Overall, this indicates that CSG-1 and CSG-3 harbor functional cGAS-STING pathway, while CSG-2 and CSG-4 are characterized by aberrant activation. Cells which are affected with high levels of CIN normally have more micronuclei that colocalize with cGAS, leading to noncanonical signaling to the NFκB p100 subunit (38). To investigate whether noncanonical cGAS-STING activation may drive CSG-2 or CSG-4, we computed the ratio between NFκB1 and NFKκ2 subunits. We observed that CSG-2 demonstrated the lowest ratio of NFκB1/NFκB2 further supporting non-canonical cGAS-STING activation (Supplementary Fig. S4D), while CSG-3 and CSG-4 both showed similar high ratios indicating canonical pathway activation. When the cGAS-STING pathway is functional, cGAS is activated through sensing of cytosolic DNA which then facilitates the production of cGAMP by catalyzing ATP and GTP and this activates STING1 (17). Thus, we would expect a positive correlation between the cGAS and STING1 genes when the pathway is active and functional. Here, we observed no correlation between cGAS and STING1 in CSG-1 and CSG-2, consistent with no activation of the cGAS-STING pathway. We see a strong correlation between cGAS and STING1 in CSG-3, indicating active pathway, while we see a negative correlation between cGAS and STING1 in CSG-4 (Supplementary Fig. S5A), indicating aberrant or nonfunctional activation. The same pattern was observed between the downstream activator genes CCL5 and CXCL10 and CXCL11, but not between CCL5 and CXCL9 (Supplementary Fig. S5B–D). Pathway inactivation may be caused by loss-of-function mutations in any pathway gene. TP53 is the most mutated cancer gene, and also plays a role in the cGAS-STING pathway. Consistent with aberrant activation of the pathway in CSG-4, we observe that this group shows the highest fraction of samples with TP53 loss of function mutations (Supplementary Fig. S6).

### CSGs Associate with Differential Activation of the Immune System

To further explore the association between the CSGs and immune cell activation, we determined for each sample the level of immune infiltration, derived from RNA-seq data as described previously (12, 36). As expected, CSG-1 and CSG-2 showed limited immune cell infiltration across all TCGA cancer types, but consistently with CSG-1 showing the least overall. Both groups CSG-3 and CSG-4 showed high levels of immune cell infiltration, with CSG-3 showing the overall highest level (Supplementary Fig. S4C), consistent with fully functional cGAS-STING pathway and the presence of TILs. Finally, we investigated the relative impact on overall survival of the CSGs. On a pan-cancer level, we found that CSG-3 was significantly associated with improved outcome whereas CSG-1, 2, and 4 all showed similar overall survival (*P* < 0.0001; Supplementary Fig. S7). In a meta-analysis for every cancer type, comparing each CSG against reference group CSG-1, we found that CSG-3 (*P* = 2.53 × 10^−6^) showed significantly improved outcome while CSG-2 showed a marginal improvement (*P* = 0.016) and CSG-4 showed increased HR however not significant (*P* = 0.111; Supplementary Fig. S8). Taken together, this demonstrates how data-driven cGAS-STING grouping associates with relevant tumor biology with potential prognostic relevance.

### cGAS-STING Pathway Activation is Associated with CIN

The cGAS-STING pathway is activated through cytoplasmic DNA and cGAS activity. Cancer CIN and genotoxic chemotherapy may both result in genomic DNA fragments leaking into the cytoplasm and activation of cGAS (19, 39–41). Thus, we hypothesized that chemotherapy combined with high levels of CIN may trigger cGAS-STING pathway activation, resulting in an antitumor immune response and improved treatment response. To investigate this hypothesis, we determined CIN using the wGII, which measures overall chromosomal alterations from the sample ploidy (37). We observed that CSG-2 generally showed higher wGII scores (*P* < 0.0001), while CSG-1 and CSG-3 showed variable distribution depending on the cancer type. CSG-4 mostly showed low wGII across all five cancer types (Supplementary Fig. S9A). Conversely, significant variation was observed between individual cancer types, indicating that CIN levels only to a lesser degree associates with CSG grouping but is highly dependent on cancer type. Similar observations were made for TMB (Fig. 2D; Supplementary Fig. S9B). A complete overview of CSG characteristics can be found in Supplementary Table S2. Finally, to investigate the prognostic impact of CSG in TCGA cohorts, we performed multivariable Cox regression, including tumor type, stage, gender, TMB, wGII, and CSG in the model (Fig. 2E). Here we observed how both wGII and CSG-3 were significant, along with tumor stage. Taken together, this demonstrates that CSG classification adds new and significant prognostic relevance in patients, even if not treated with immunotherapy.

### CSG Classification Distinguish Between Types of Immune Cell Infiltration

**FIGURE 3:**
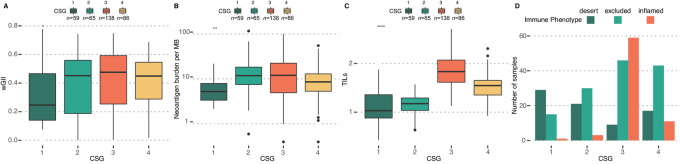
CSGs in the BLCA immunotherapy cohort. Boxplots of CSGs versus wGII (**A**), neoantigen burden (**B**), and TILs (**C**). CSG-3 shows increased levels of all measures. **D,** Barplot showing immune phenotype defined by IHC versus CSG, demonstrating high enrichment of inflamed immune phenotype in CSG-3.

Having established a prognostic role of and a plausible link between wGII and CSGs, we investigated the significance in an immunotherapy-treated cohort. For this analysis, we acquired a dataset of 348 patients with metastatic BLCA (31) treated with the anti-PD-L1 agent atezolizumab and analyzed using RNA-seq and WES of the tumor, and IHC for TILs (a complete overview of CSG characteristics in this cohort can be found in Supplementary Table S3). Here, we observed the highest level of wGII in CSG-3, while CSG-2 contained the highest level of neoantigens (Fig. 3A and B). When we investigated the overall TIL level from RNA-seq, we found that CSG-3 showed the highest level followed by CSG-4 (Fig. 3C). However, when we compared the TIL infiltrate between CSGs using IHC data, we observed that CSG-3 was enriched in the “inflamed” phenotype, indicating immune cells infiltrating the carcinoma cells, while CSG-4 was highly enriched in the “excluded” and “desert” phenotypes, indicating immune cells not infiltrating the carcinoma cells. These results suggest distinct types of immune cell activation between CSG-3 and CSG-4, which are not identified through total TIL estimates alone (Fig. 3D).

### CSG Associates with Response to Immunotherapy

**FIGURE 4:**
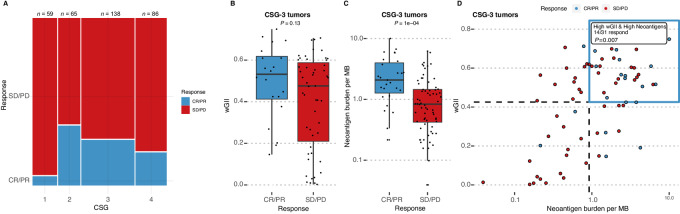
CSG-3 shows improved response to immunotherapy in the BLCA immunotherapy cohort. **A,** Mosaic plot showing immunotherapy response varies by CSG, with CSG-1 showing very poor response. Bar width is proportional to the number of patients within each CSG. **B,** Boxplot showing wGII versus response within CSG-3 samples. **C,** Boxplot showing neoantigen burden versus response within CSG-3 samples. **D,** Scatterplot of wGII and neoantigen burden. Each value is divided into high and low by the median as shown by dotted lines. Blue quadrant demarcates samples with high-wGII and high-neoantigens. These patients show improved response relative to all others, with 45% of all CSG-3 patients with high-wGII and high-neoantigens showing response to immunotherapy. CR: Complete response. PR: Partial response. SD: Stable disease. PD: Progressive disease.

Next, we investigated the association between CSG groups and immunotherapy response. Analyzing binary response data where positive outcome is defined as complete response and partial response, we observed that CSG-2 and CSG-3 showed the highest percentage of responders (36% and 27%, CSG-2 and CSG-3, respectively, *P* < 0.001), while CSG-1 showed the worst response, with only 3 of 56 (5%) patients showing an objective response (Fig. 4A). Within CSG-3, patients that responded to immunotherapy showed higher wGII (mean 0.499 vs. 0.399, *P* = 0.13; Fig. 4B) and significantly increased neoantigen burden (mean 2.82 vs. 1.25, *P* < 0.0001; Fig. 4C), indicating an interaction between CSG, CIN, neoantigen burden, and immunotherapy response. To further investigate this, we split the CSG 1–4 tumors into high and low wGII and high and low neoantigen burden based on the median. We observed that patients with CSG-3 tumors with high wGII and high neoantigen burden showed increased response relative to all other groups (Fig. 4D; *P* = 0.007). Interestingly, while weaker, this pattern was also observed within CSG-4 (*P* = 0.024; Supplementary Fig. S10). No significant association was observed for CSG-1 (*P* = 1) and CSG-2 (*P* = 0.49). When we compared the correlation between activator and response genes in the BLCA immunotherapy cohort, we observed CSG-4 showed no correlation between gene sets, while CSG-1, -2, and -3 did. CSG-3 and CSG-4 showed similar expression of activator genes; however, CSG-4 showed reduced expression of response genes (Supplementary Fig. S11). CSG-S2 showed low levels of both activators and response gene sets, indicating a nonfunctional cGAS-STING pathway. This is consistent with CSG-3 harboring functional and active cGAS-STING signaling, where CIN results in a stronger immune response when stimulated by checkpoint immunotherapy. In turn, the immune response is more likely to be sustained when the cancer cells harbor a high level of neoantigens that may be recognized by infiltrating immune cells. CSG-4 tumors appear to harbor a functional cGAS-STING pathway that does not activate. Given that both CSG-2 and CSG-4 also show response to immunotherapy, the immune response may be mediated through non-cGAS-STING mechanisms in these groups.

### CSG Identifies Patients with Improved Prognosis

**FIGURE 5:**
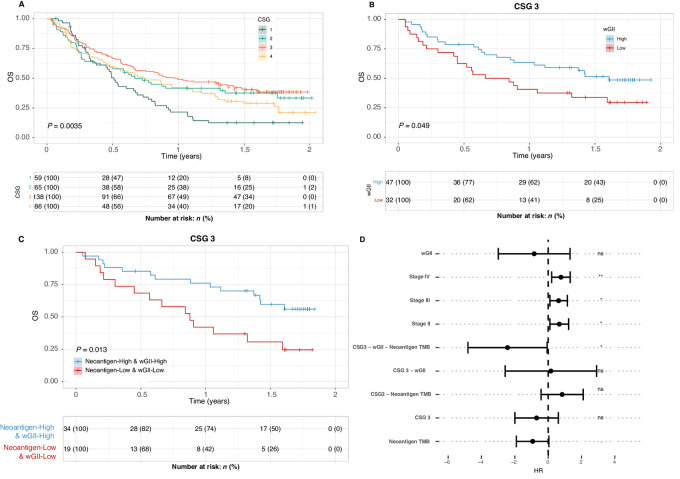
CSG-3 shows improved survival following immunotherapy in the BLCA immunotherapy cohort. **A,** Kaplan–Meier analysis showing CSG versus overall survival, demonstrating particularly poor outcome for CSG-1. **B,** Kaplan–Meier analysis of CSG-3 tumors, with tumors split into high and low CIN based on median wGII score of the whole cohort. **C,** Kaplan–Meier analysis of CSG-3, comparing the high-neoantigen burden, high-wGII group to the low-neoantigen, low-wGII group. **D,** Multivariate Cox proportional hazards model including CSGs, wGII, neoantigens, stage, and TILs, including a test for the interaction between CSG, wGII, and neoantigens. This shows significant interaction between CSG-3, wGII, and neoantigens, associating with improved outcome. The full model parameters and corresponding estimates can be found in Supplementary Table S4.

When we analyzed overall survival time in the immunotherapy-treated cohort, we found that CSG-2, -3, and -4 demonstrated similar survival with CSG-3 being slightly better. CSG-1 on the contrary showed significantly reduced overall survival, with a median survival of only 6 months compared with 8, 12, and 9 for CSG-2, -3, and -4 (Fig. 5A). Overall, this indicates that CSG classification alone is informative with regard to overall survival following immunotherapy. Next, we asked whether inclusion of the wGII measure would impact prognosis consistent with the hypothesis that increased CIN in the context of an active cGAS-STING pathway would improve the immune response. We split the cohort into high and low CIN based on the median wGII score. We then compared overall survival within each CSG group between high and low CIN. We observed a clear split within CSG-3, showing that patients with high CIN tumors had improved survival when treated with immunotherapy (*P* = 0.049, Fig. 5B; Supplementary Fig. S12A–S12D). Checkpoint immunotherapy is hypothesized to boost an adaptive immune response driven by cytotoxic CD8 T cells targeting cancer neoantigens. As such, previous work has shown how an increase in cancer neoantigens is associated with improved response in urothelial cancer (42). We further hypothesize that tumors with high CIN and functional cGAS-STING pathway can mount a more effective immune response, but that an adaptive CD8 response still requires cancer neoantigens. Thus, high-CIN, high-neoantigen tumors with functional cGAS-STING should show improved response and extended survival time. To test this, we defined tumors as high and low neoantigens based on the median value, and asked whether high-wGII and high-neoantigens tumors may show improved prognosis and higher frequency of responders. Indeed, within CSG-3, tumors with both high-wGII and high-neoantigens showed superior outcome (*P* = 0.013; Fig. 5C). Finally, we performed multivariate Cox regression against overall survival including interaction between CSG, wGII, and neoantigens. The model showed that CSG-3, wGII and neoantigen burden all independently showed negative HR. However, when combined the interaction represents a significantly improved outcome (HR = 0.09, *P* = 0.04; Fig. 5D). The full model parameters and corresponding estimates can be found in Supplementary Table S4. These results demonstrate how we can identify a subset of patients with active cGAS-STING signaling, where high CIN associates with improved outcome to immunotherapy. Overall, this indicates that response to immunotherapy may associate with elements of the innate and adaptive immune system and is affected by both cancer-intrinsic CIN and cancer neoantigens.

## Discussion

In this work, we explored the role of the cGAS-STING pathway and cancer-induced CIN in the context of immunotherapy response. By defining cGAS-STING activation status on a pan-cancer level using genes with known roles in the pathway, we were able to build a gene expression–based model of cGAS-STING activation based on unsupervised clustering. This model is represented by four distinct classes likely reflecting different levels of activation of the innate immune system. Each class demonstrates different overall survival time and in the context of immunotherapy shows different levels of response. Using a random forest model, made available with this publication, each class can be reliably recovered, making our CSG classification system suitable for single-sample use. The cGAS-STING pathway is activated through cytosolic DNA, a common by-product of CIN (16, 17). Previous work has demonstrated how the cGAS-STING pathway is commonly activated by DNA from micronuclei bursting in the cytosol in tumors with high levels of CIN (43). On its own, high levels of CIN have previously been shown to be associated with poor outcome (44, 45). However, when we analyzed CIN, as defined by the wGII score within CSG classes in patients treated with immunotherapy, we observed that patients with high wGII is associated with improved outcome, but only in CSG-3, the groups likely harboring active cGAS-STING pathway (Supplementary Fig. S12). This supports a model where CIN in tumors with active cGAS-STING pathway creates an inflamed local environment with increased immune cell infiltration of the carcinoma cells. Previous work (31, 46) has demonstrated how IHC may classify human tumors into immune-inflamed (immune cells infiltrating the carcinoma cells), immune-excluded (immune cells found only at the tumor periphery), and immune-desert (no immune cell association with tumor) phenotypes. Of these, the immune-inflamed phenotype is particularly associated with response to immunotherapy (46). Interestingly, while both CSG-3 and CSG-4 showed high levels of TILs as summarized by RNA-seq (Fig. 2D and Fig. 3C), tumors with a CSG-3 profile were commonly of the immune-inflamed phenotype. Consistent with this observation, we found CSG-3 to be dominant within the cancer types known to be enriched in high mutation burden (Fig. 2B). Conversely, CSG-4 tumors were predominantly of the immune-excluded phenotype, where the immune cells remained in the periphery (31). In the non—immunotherapy-treated TCGA dataset, we observed that CSG-4 was associated negatively with outcome (Supplementary Fig. S8), yet in the BLCA immunotherapy-treated Mariathasan cohort, we observed a subset of patients with good response (Supplementary Fig. S10). Here, despite a likely inactive cGAS-STING pathway as defined on the basis of activator and response gene sets, we observed a significant increase in both wGII and neoantigen burden among patients responding well to therapy, indicating that while CSG-4 is characterized by poor prognosis and aberrant cGAS-STING activation (Supplementary Fig. S11), checkpoint immunotherapy may be beneficial to a subset of patients in this group. While not all tumors with high TMB show response to immunotherapy, it is well established that an association exists between TMB and immunotherapy response (47). TMB is likely a proxy measure of cancer neoantigens, which represent the targets of the adaptive anticancer immune response (48), and as such are likely a requirement for response to immunotherapy. We observed that CSG-2, CSG-3, and CSG-4 all contained tumors with high levels of wGII and neoantigens, yet most immunotherapy responders were found within CSG-3. In addition, in a multivariate model, we found a significant interaction between CSG-3, wGII, and neoantigen burden.

Our work thus supports that a link exists between the innate and the adaptive immune response, where cancer-associated CIN occurring in cancers with functional cGAS-STING pathway results in activation of an IFN response and recruitment of immune cells to the local tumor microenvironment. As the primary tumor evolves, it develops alternative immune-evasion mechanisms to avoid immune-mediated destruction. When treated with immunotherapy, these mechanisms may be overcome and an adaptive immune response directed against cancer neoantigens may result in a strong and sustained anticancer response with improved long-term durability. Taken together, our findings indicate that combining biomarkers of cGAS-STING activity, CIN, and neoantigen burden may improve response prediction to immunotherapy and potentially broaden the score of eligible patients by identifying small subsets of highly sensitive patients within cancer histologies that commonly fail to respond to immunotherapy. While further research in this field is required to properly assess the utility of these biomarkers, this work demonstrates a plausible link between CIN and immunotherapy response, dictated by the activity of the innate immune response. However, this should be further validated in additional cohorts. We have provided a gene expression–based biomarker to assist with defining the activity of the cGAS-STING pathway, available to the community as a ready to use R script but further work must be performed to validate the different classes of immune activation relative to immunotherapy response across multiple cancer types.

## Supplementary Material

Supplementary Data Supplementary data
