# Supplementary material for: Classifying cGAS-STING Activity Links Chromosomal Instability with Immunotherapy Response in Metastatic Bladder Cancer
Source: Cancer Res Commun. 2022 Aug 4;2(8):762–71. doi: 10.1158/2767-9764.CRC-22-0047 (PMC10010288; doi:10.1158/2767-9764.CRC-22-0047)
Supplement: Supplementary Data [file crc-22-0047-s01.pdf]

Supplement Material for

Classifying cGAS-STING activity links chromosomal instability with immunotherapy response in metastatic bladder cancer

Supplementary Table 1	cGAS-STING interacting genes
Supplementary Table 2	Table showing molecular characteristics of CSG1-4 in TCGA
Supplementary Table 3	Table showing summary of identified CSG in the BLCA immunotherapy cohort
Supplementary Table 4	Table shows coefficients on BLCA immunotherapy cohort based on cox proportional hazard multivariate model
Supplementary Figure 1	Page 5
Supplementary Figure 2	Page 6
Supplementary Figure 3	Page 7
Supplementary Figure 4	Page 8
Supplementary Figure 5	Page 9
Supplementary Figure 6	Page 10
Supplementary Figure 7	Page 11
Supplementary Figure 8	Page 12
Supplementary Figure 9	Page 13
Supplementary Figure 10	Page 14
Supplementary Figure 11	Page 15
Supplementary Figure 12	Page 16

SUPPLEMENTARY TABLES

Supplementary Table 1: cGAS-STING interacting genes

Gene	Reference
STING1	[16], [17], [19], [28], [30], [48], [37], [18]
NFKB1	[16],[17],[28],[30],[37],[42]
IKBKG	[48]
TRAF6	[37,49]
RELA	[16],[17],[28],[30],[37]
NFKB2	[16],[17],[28],[37],[42],[48]
RELB	[16],[17],[28],[48],[37],[42]
MAP3K14	[18, 48]
CCL5	[19]
CXCL9	[16,19]
CXCL10	[16,19]
CXCL11	[19]
CGAS	[16],[17],[19],[28],[30]
ENPP1	[28]
TREX1	[37,48,49]
IFI16	[28],[48]
IRF3	[16],[17],[28],[30],[37],[49]
ATM	[18, 48]
TBK1	[17],[28],[30],[48],[37],[18]
PARP1	[18, 48]

Supplementary Table 2: Table showing the molecular characteristics of CSG1-4 in TCGA.

CSG	n	wGII	TMB	Responder genes	Activator genes	Responders-Activators ratio	Interpretation
**1**	361	0.41	4.58	0.04	3.62	0.001	Inactive
**2**	309	0.51	3.51	2.12	2.93	0.723	Non-functional
**3**	1048	0.46	5.16	5.16	4.41	1.17	Active
**4**	589	0.47	3.22	2.98	4.28	0.69	Abnormal activation

Supplementary Table 3: Table showing the summary of identified CSG in the BLCA immunotherapy cohort

CSG	n	wGII	TMB	Responder genes	Activator genes	IO Responders
**1**	59	0.31	0.66	0.50	2.86	3
**2**	65	0.39	1.68	0.93	2.49	18
**3**	138	0.42	1.73	2.55	3.25	33
**4**	86	0.39	1.19	1.31	3.16	14

Supplementary Table 4: Table showing estimated coefficients, their confidence intervals, hazard ratio and p-value of multivariate cox-proportional hazard model in immunotherapy treated cohort

	Pr(>|z|)	2.5 %	97.5 %	coef	exp(coef)
**Stage II**	**0.0203403748937784**	0.103194193813057	1.22670227074922	0.664948232281138	1.94438986210367
**Stage III**	**0.0190951008239468**	0.102769423905358	1.15266075046287	0.627715087184113	1.87332530039723
**Stage IV**	**0.00691135643931658**	0.210104970967461	1.32138876353428	0.765746867250868	2.15059998769121
**CSG2**	0.441871651781437	-1.96638443993066	0.85812418536040	-0.554130127285132	0.57457184824988
**CSG3**	0.297427390259272	-1.99564813060135	0.61027428260066	-0.692686924000341	0.50023018124695
**CSG4**	0.246936364549537	-0.60227020207462	2.34092209479392	0.869325946359645	2.3853024896969
**wGII**	0.444850517018845	-2.99078018926334	1.31306603791549	-0.838857075673923	0.43220421796213
**TMB**	0.0613319062308628	-1.90824464846835	0.04426547546405	-0.931989586502148	0.39376949202887
**TIL**	0.428140881579202	-1.05225883130894	0.44638499035084	-0.302936920479048	0.73864568831714
**Gender = Male**	0.864794010288844	-0.44893403915198	0.534359112728211	0.0427125367881154	1.04363784423917
**CSG2 - wGII**	0.911691703472188	-4.36608338041723	3.89843394249033	-0.23382471896345	0.79150053877235
**CSG3 - wGII**	0.907236972883025	-2.57797550547985	2.90388471381611	0.162954604168133	1.17698325834159
**CSG4 - wGII**	0.162014266915642	-7.3902653073123	1.23593468638121	-3.07716531046555	0.04608972169947
**CSG2- TMB**	0.126749414736743	-0.2335965136380	1.88154512643393	0.823974306397942	2.27954145474069
**CSG3 - TMB**	0.19361621395722	-0.42592427142464	2.10372884430321	0.838902286439286	2.3138256644108
**CSG4 - TMB**	0.382146243489956	-4.08394418534222	1.56505398657063	-1.25944509938579	0.28381146997199
**wGII - TMB**	**0.0191915532513094**	0.363234549626664	4.09230579524581	2.22777017243624	9.27915208466129
**CSG2 - wGII- TMB**	0.0701265343540397	-5.02205283119336	0.19825756127655	-2.4118976349584	0.08964501954049
**CSG3 - wGII- TMB**	**0.0443720743209857**	-4.81381779544736	-0.06134065225412	-2.43757922385074	0.0873721039666
**CSG4 - wGII - TMB**	0.531132206789143	-4.42498082079392	8.58078991890764	2.07790454905686	7.98771350356211

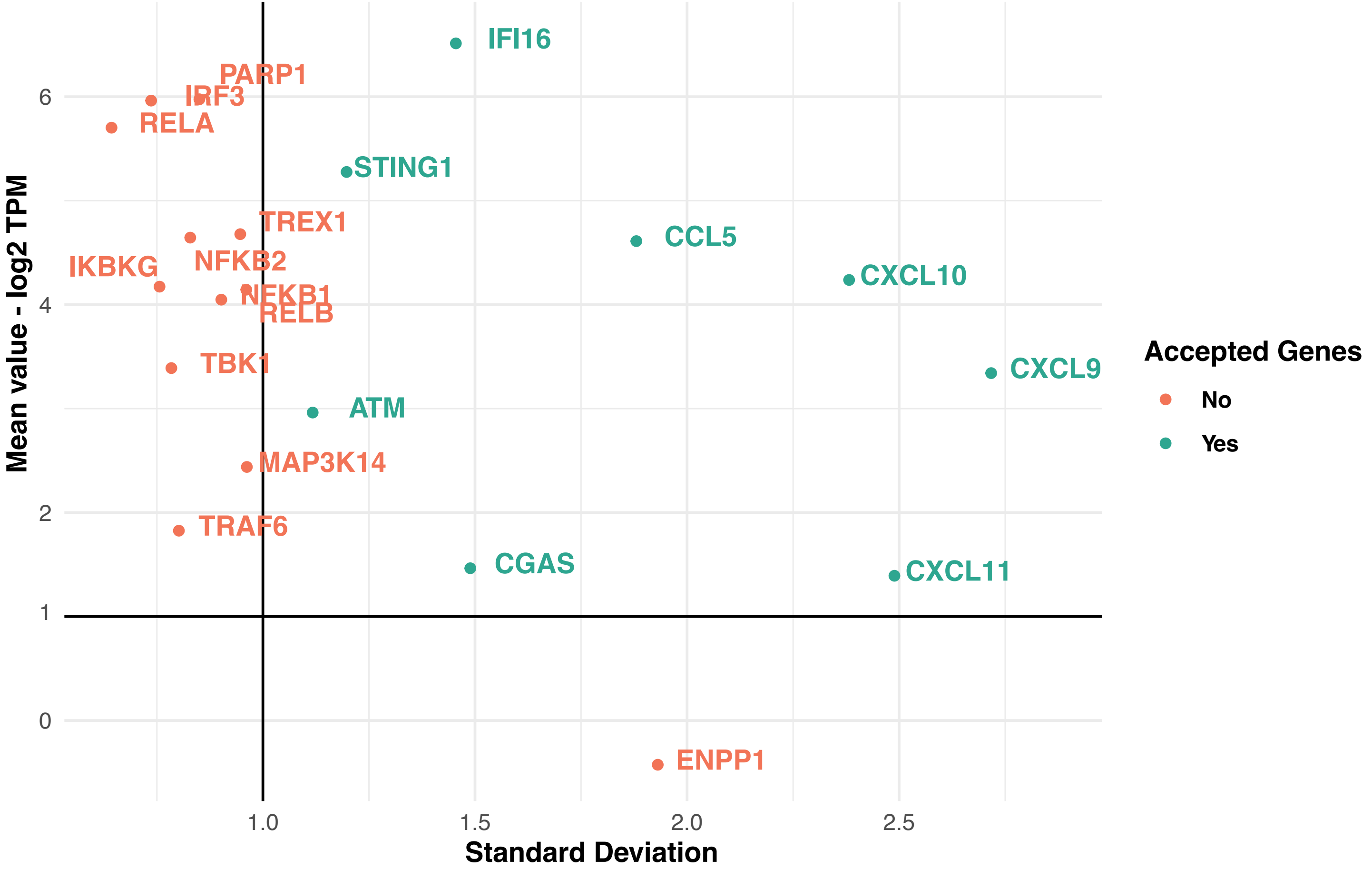

Figure S1. Scatter plot showing mean gene expression of the selected immune genes across the chosen cancer types (TCGA) versus the standard deviation. Values of "Yes" were used to identify genes with sufficient expression and high inter-sample variation.

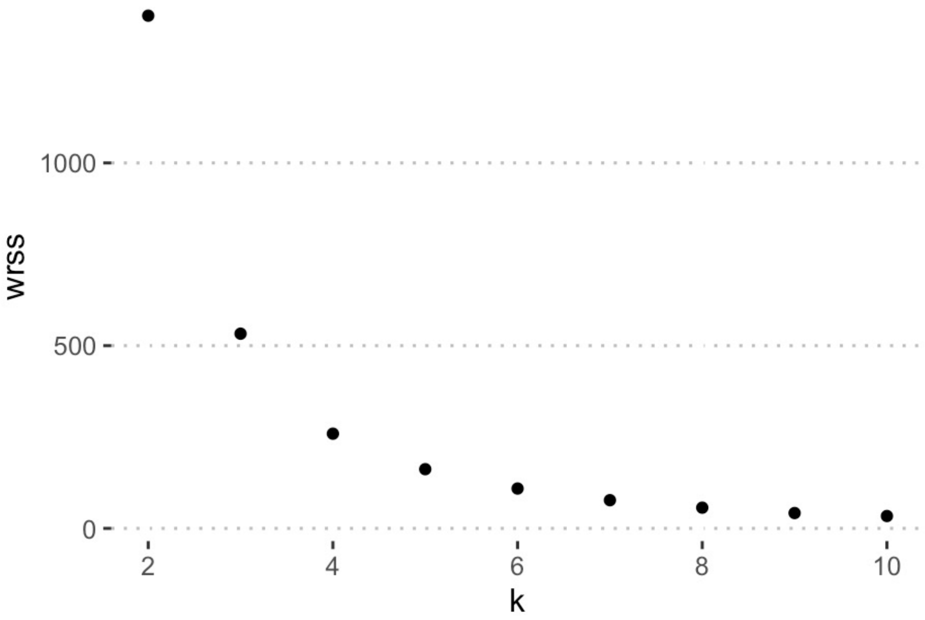

Figure S2. K-means elbow plot, showing Within-Cluster-Sum of Squared Errors (WSS) versus increasing values of K. Optimal number of clusters found at k= 4.

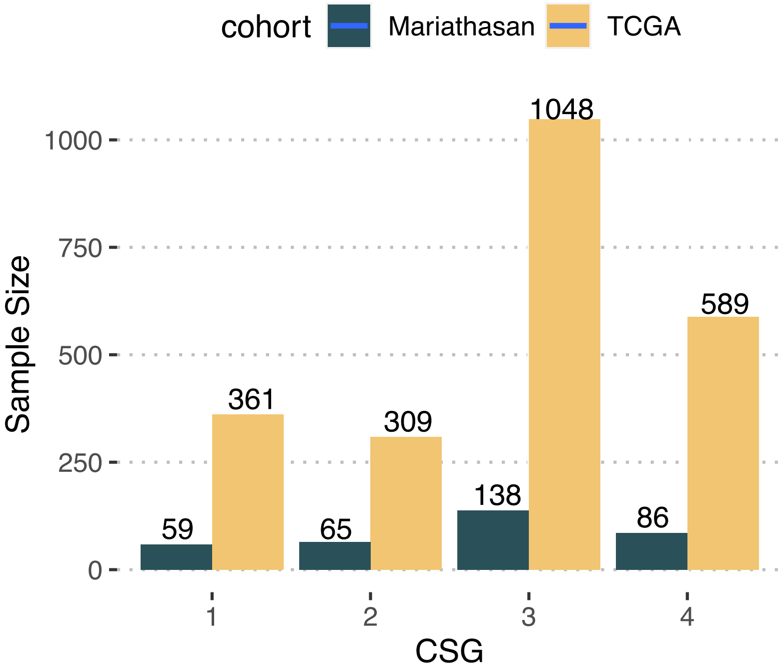

Figure S3. Sample sizes corresponding to each CSG and each cohort.

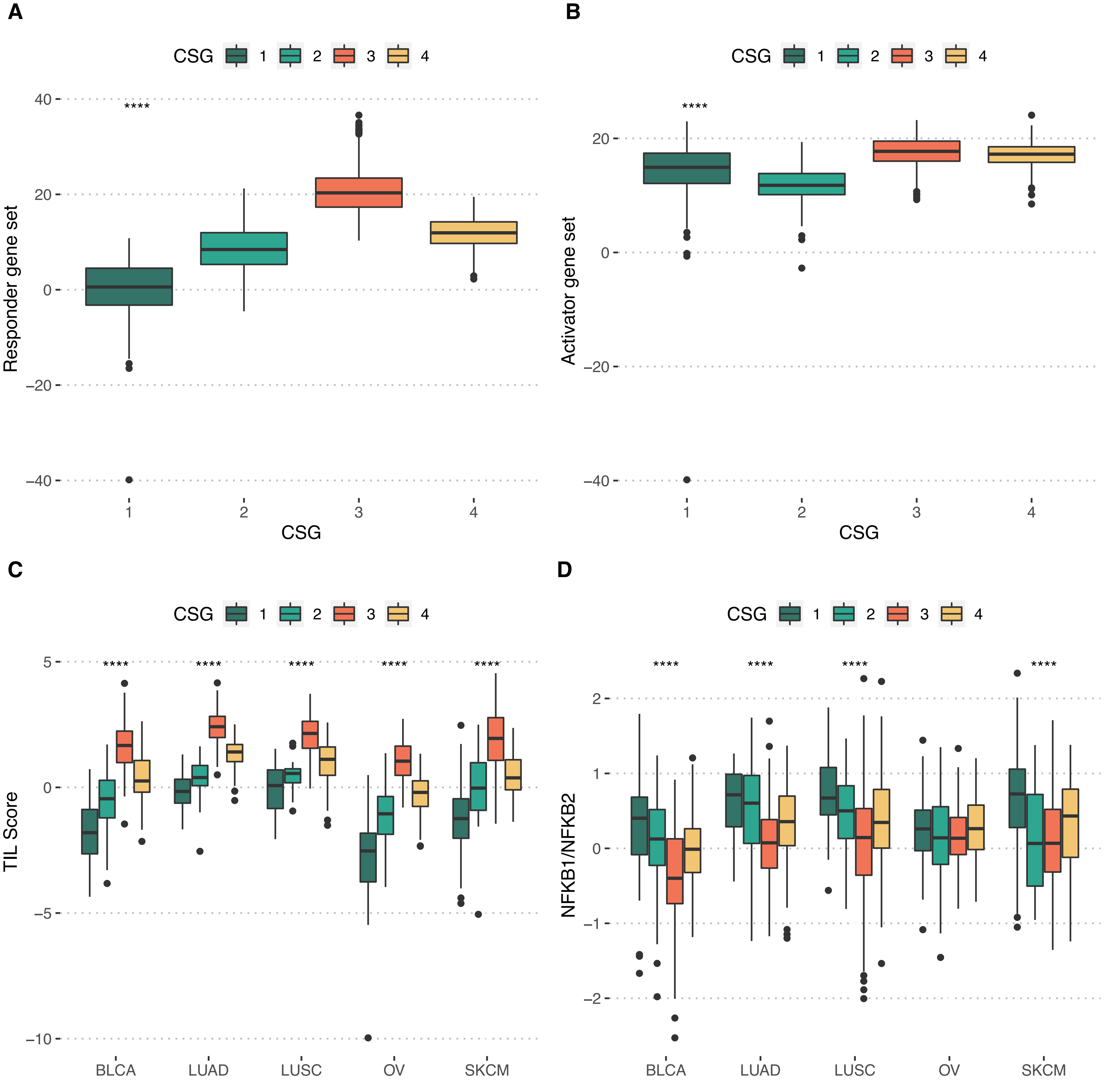

Figure S4. Mean expression of Activator (A) and Responder (B) genesets relative to CSG. (C) TIL score by CSG and by cancer type. (D) Gene expression ratio between NFKB1 and NFKB2 by CSG and by cancer type.

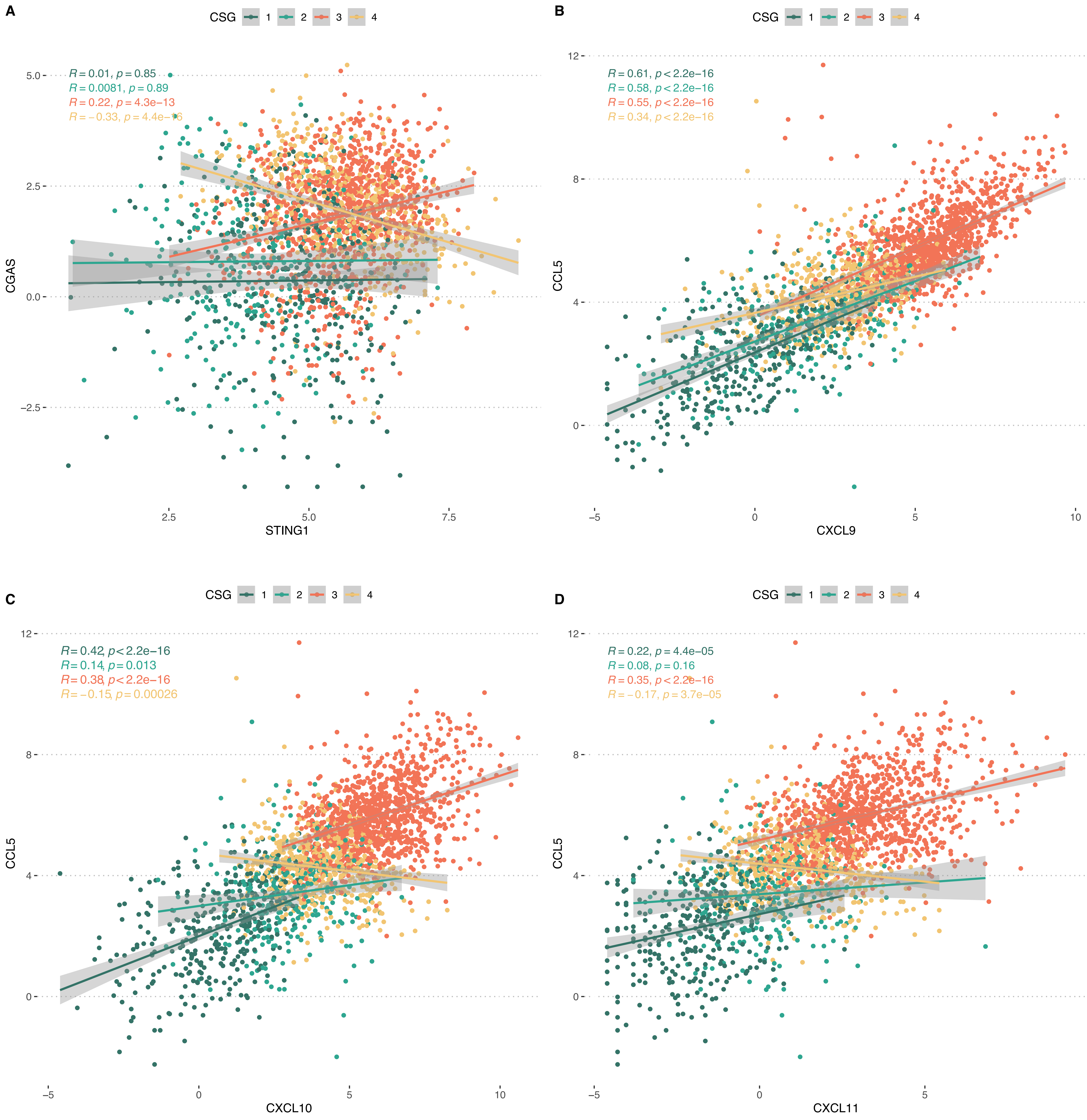

Figure S5. Scatter plots showing different correlation depending on CSG class between (A) Activator genes cGAS and STING1 within CSG 1–4, (B–D) Responder genes CCL5 and CXCL9, CXCL10 and CXCL11.

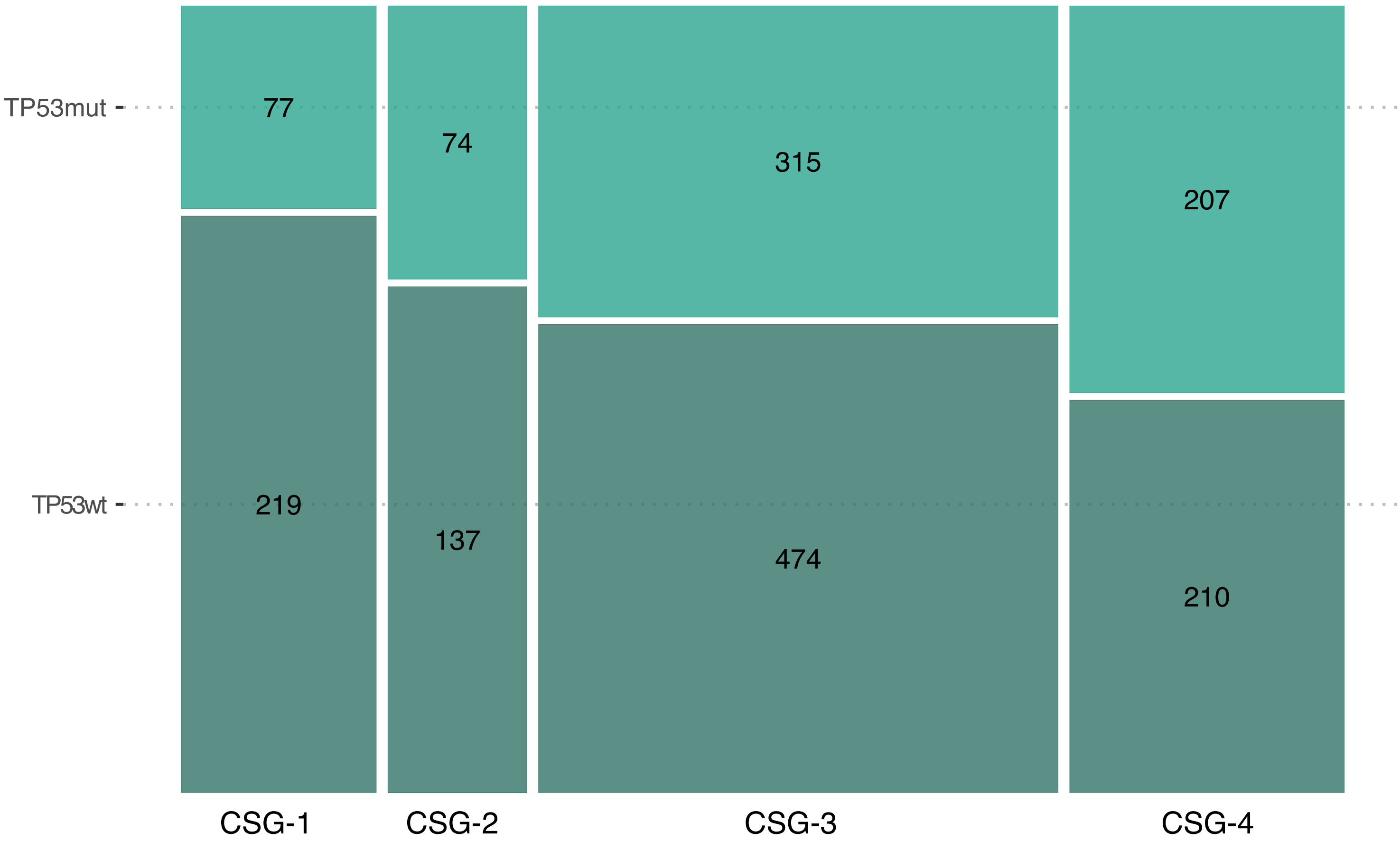

Figure S6. Distribution of samples with TP53 mutation by CSG across 2307TCGAtumours

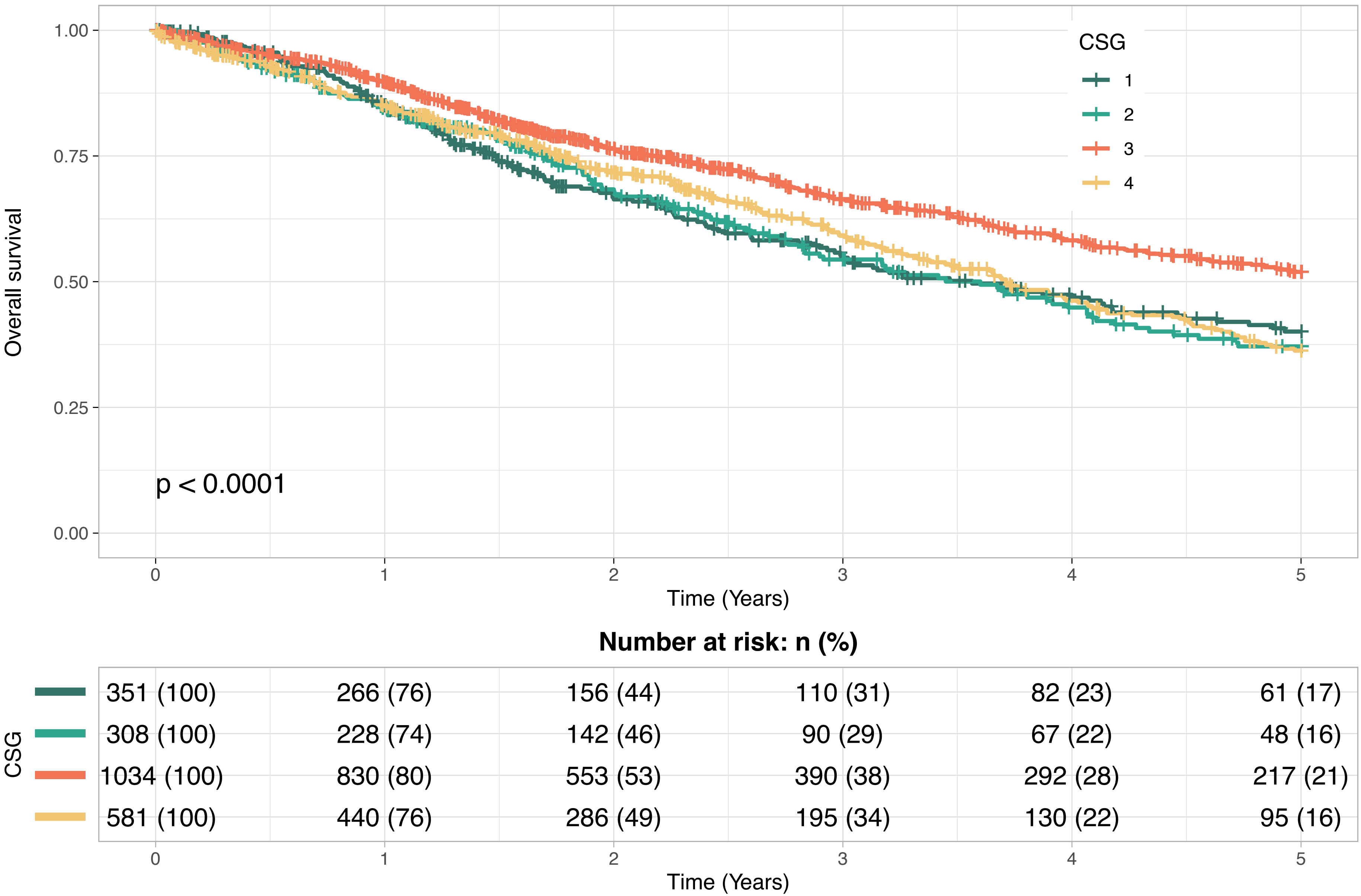

Figure S7. Overall survival time within the combined 2307 TCGA samples representing patients with bladder, lung adeno, lung squamous cell, ovarian and melanoma cancer when stratified by CSG.

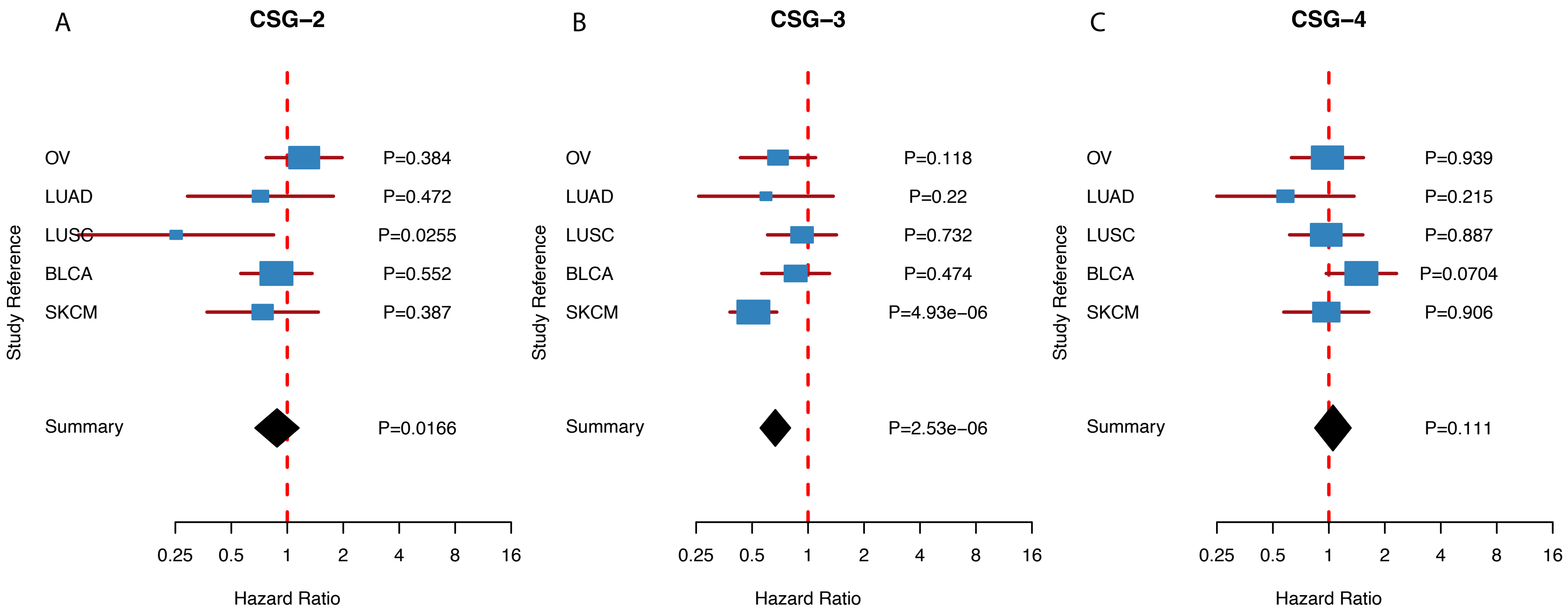

Figure S8. Meta-analysis of over-all survival time across bladder (BLCA), lung adeno (LUAD), lung squamous cell (LUSC), ovarian (OV), and melanoma (SKCM) samples, comparing CSG-1 against (A) CSG-2, (B) CSG-3, (C) CSG-4, respectively. This shows CSG-3 strongly associates with improved outcome. P-values for individual cancer types are based on Cox proportional hazard models. Summary P-value is calculated based on Fisher's method.

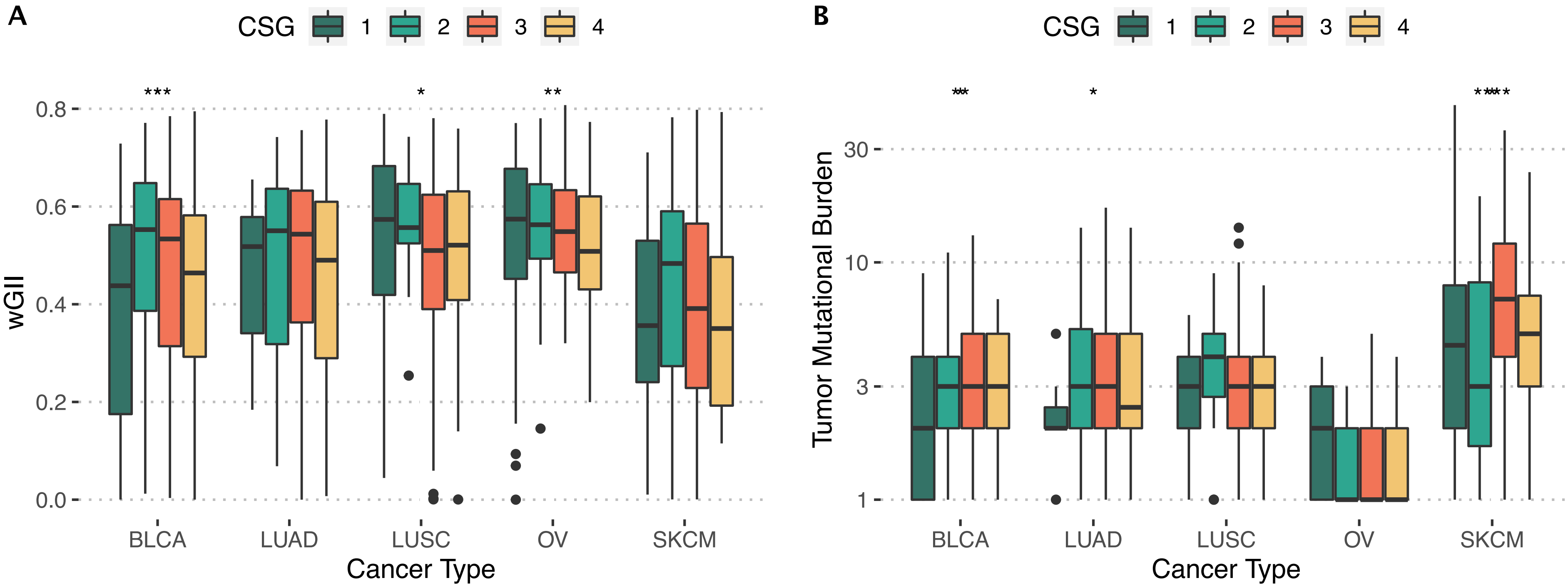

Figure S9. Distribution of (A) wGII and (B) tumour mutation burden by CSG and cancer type.
* indicates $p < 0.05$ within a given cancer type

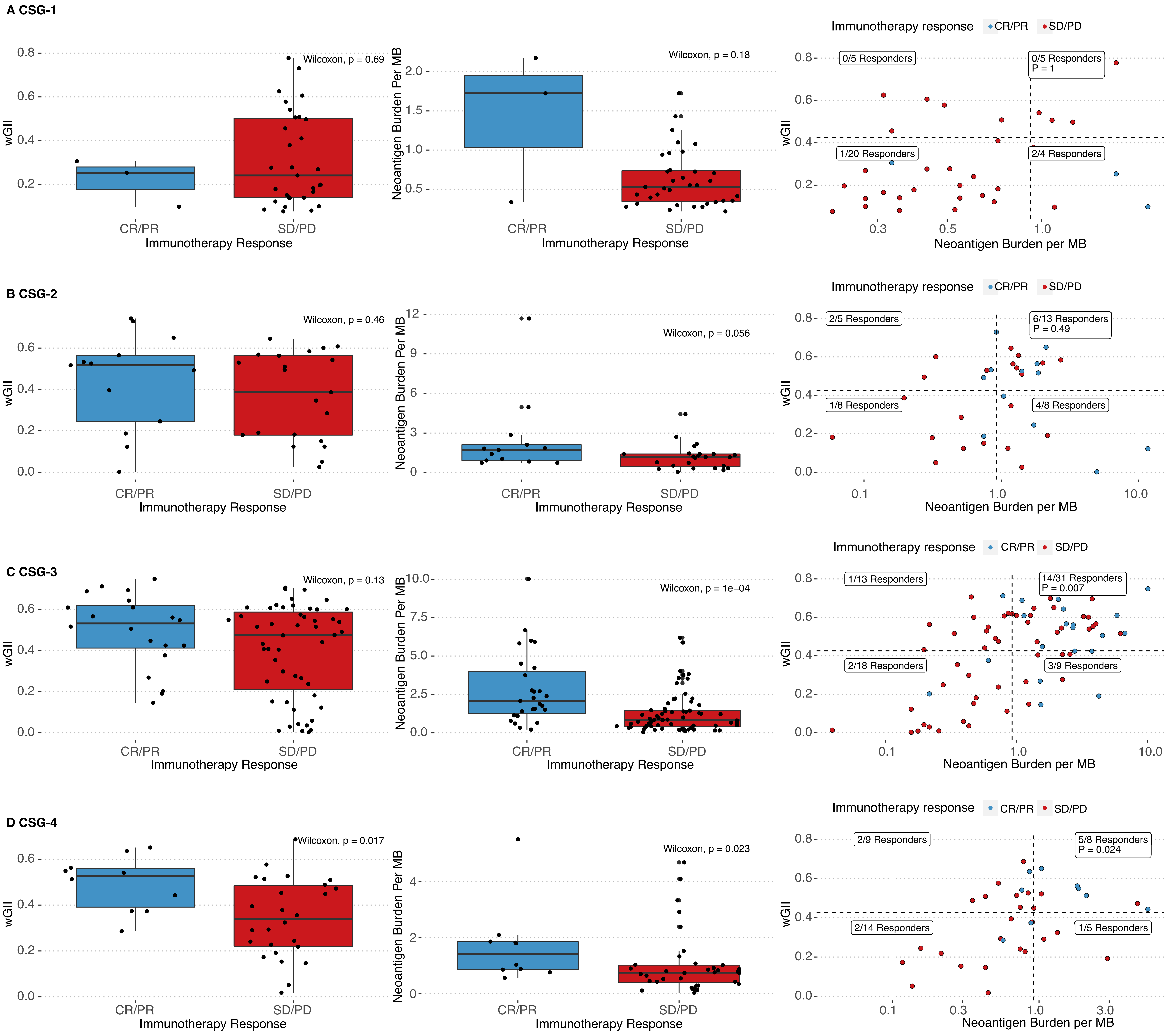

Figure S10. Immunotherapy response in the validation cohort, shown within CSG−1(A), CSG−2(B), CSG−3(C) and CSG−4(D). Each row shows binary therapy response against wGII and Neoantigen burden, and a scatter plot showing wGII versus neoantigen burden.
CR: Complete response. PR: Partial response. SD: Stable disease. PD: Progressive disease.

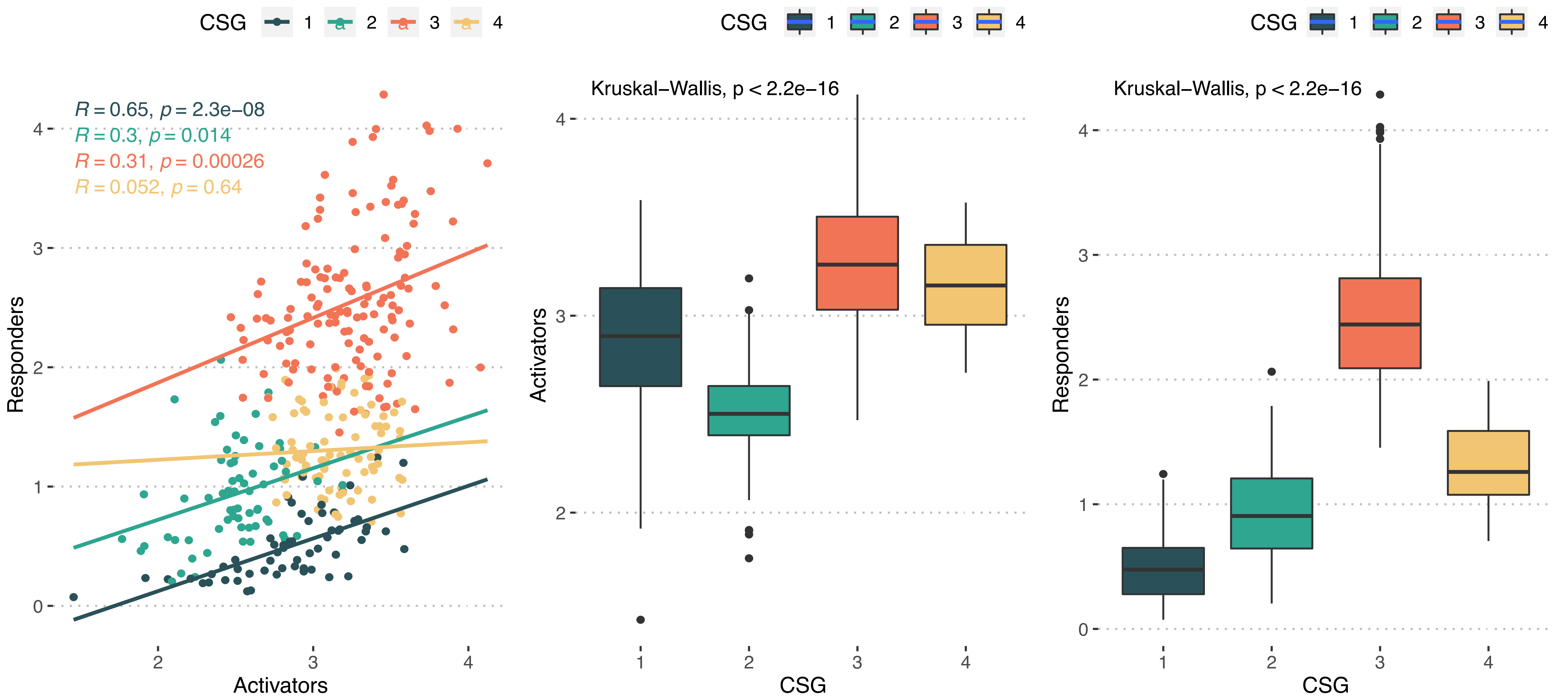

Figure S11. Analysis done on the test cohort indicates the difference between CSG-3 and CSG-4. CSG-3 shows positive correlation between mean expression of activator genes and mean value of responders genes (A). CSG-3 and CSG-4 show similar patterns in expression of activator genes (B) however responder genes are significantly lower in CSG-4 (C)

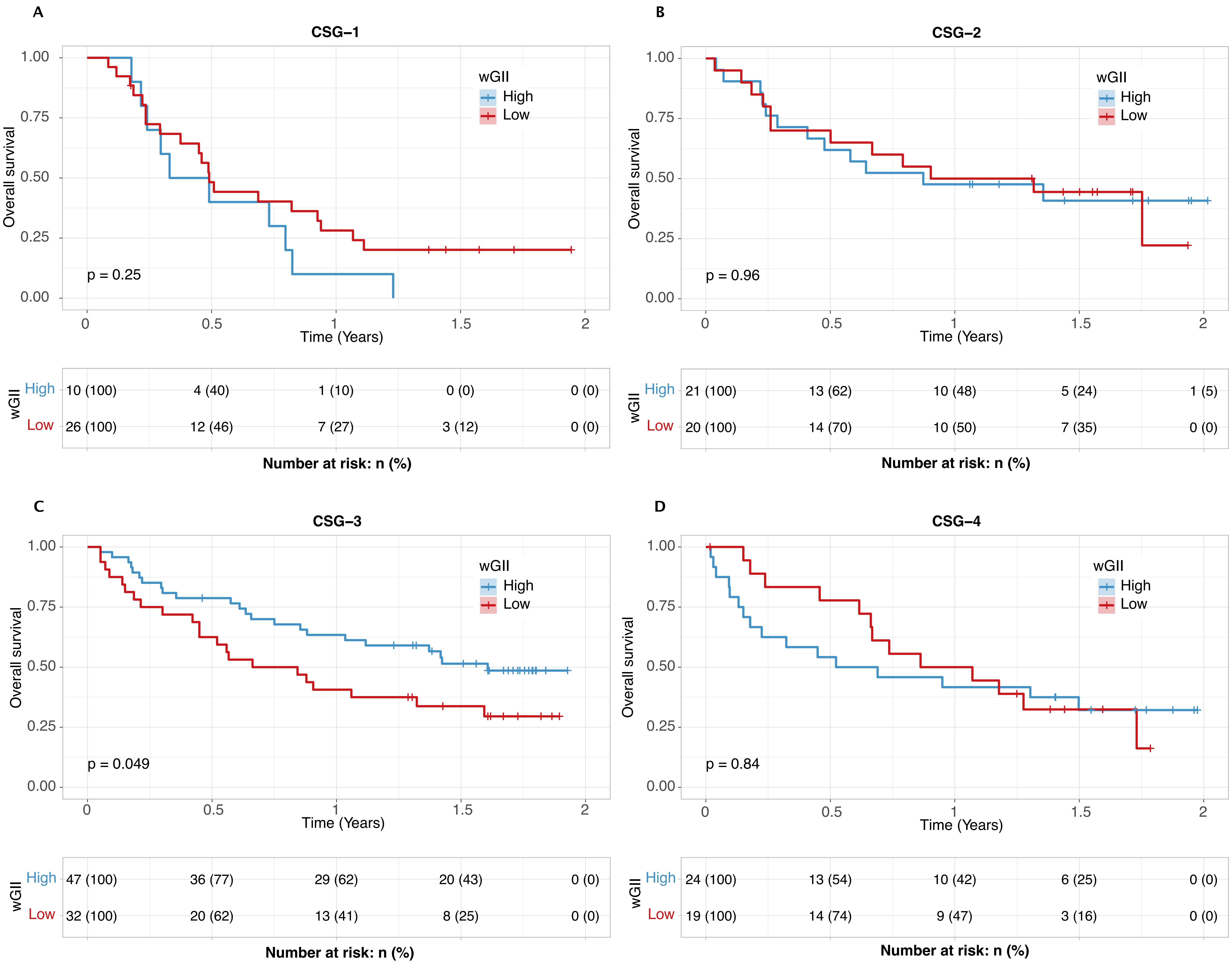

Figure S12. Kaplan–Meieranalysis of the validation cohort, showing overall survival by CSG 1–4when split into high and low wGII based on the median value of the cohort.
